# Creating healthy organizations: an integrated culture of care in high-demand sectors—a qualitative study of private-sector organizations in Sweden

**DOI:** 10.3389/fpubh.2026.1888516

**Published:** 2026-09-16

**Authors:** Åsa Stöllman, Fredrik Molin, Kristina Eliasson

**Affiliations:** 1 Department of Medical Sciences, Occupational and Environmental Medicine, Uppsala University, Uppsala, Sweden; 2 Department of Occupational and Environmental Medicine, Uppsala University Hospital, Uppsala, Sweden; 3 IPF, The Institute for Organizational and Leadership Development, Uppsala University, Uppsala, Sweden

**Keywords:** ethics of care, health and safety culture, health-promoting leadership, occupational health and safety management, organizational health factors, organizational level health-promotion, psychosocial work environment, sustainable work environments

## Abstract

**Introduction:**

“High-demand sectors” are characterized by substantial physical, psychological, and emotional demands, alongside persistent recruitment and retention challenges. Despite growing interest in shaping healthy organizations, research has largely focused on workplace- or individual-level determinants. The aim of this study was to deepen the understanding of organizational-level health-promoting factors by exploring experienced managers’ perspectives and visions regarding the promotion of a healthy work environment in high-demand sectors.

**Methods:**

Using an exploratory qualitative design, semi-structured digital interviews were conducted with 16 managers from 15 organizations in the transport, health and social care, construction, and retail trade sectors. The data were analyzed using an inductive-to-abductive thematic analysis informed by relevant theory on organizational health-promoting factors.

**Results:**

The findings indicated that healthy organizations are understood not as products of isolated wellbeing initiatives, but as organizations characterized by an integrated culture of care embedded in structures, routines, and leadership practices. Four interrelated themes were identified: integrated occupational health and safety management system, strategic efforts to promote organizational attractiveness across the employee lifespan, present and supportive leadership focused on employee wellbeing, and effective communication structures. Managers emphasized systematic and participatory occupational health and safety practices, structured onboarding, competence development, flexible accommodation across the employment life course, visible and relationally close leadership, and open, psychologically safe communication as central organizational resources for balancing high demands and sustaining both employee wellbeing and organizational functioning.

**Discussion:**

This study extends prior research on healthy organizations by highlighting the importance of a lifespan-oriented workforce strategy and by framing an organization-wide culture of care as a unifying condition for resilience, retention, safety, and long-term work ability in high-demand sectors.

## 1 Introduction

Creating and sustaining healthy organizations is a central priority in occupational health research and practice (1, 2). Research has shown that healthy organizations do not emerge by chance, but through deliberate efforts to create conditions that support both individual wellbeing and organizational performance (3). Consequently, research has increasingly shifted from a traditional focus on risks and ill health toward understanding the characteristics of healthy workplaces—settings in which employees thrive, develop, and maintain low levels of sickness absence while achieving organizational goals (4–7).

Developing healthy organizations depends on improving working conditions and organizational structures. Consistent with the hierarchy of controls in occupational safety and health, this involves prioritizing organizational-level measures that reduce or eliminate risks to employee health and wellbeing, rather than relying primarily on individual-level interventions (8, 9). Consequently, there is a clear need to expand our understanding of organizational-level health-promoting factors that can effectively enhance employee wellbeing. Globalization, digitalization, efficiency pressures, and demographic shifts, such as rising retirement ages, are reshaping working life (10). These changes underscore the importance of creating age-inclusive, attractive, and sustainable workplaces that support long-term work ability across the lifespan.

Several sectors stand out because of their combination of high job demands, high turnover, and persistent recruitment difficulties. These so-called “high-demand sectors” are characterized by substantial physical, psychological, or emotional pressures, including heavy workloads, time constraints, safety risks, and emotionally challenging interactions. If not balanced by adequate organizational resources, such demands increase the risk of stress, burnout, injuries related to physical strain, and other work-related health problems. Additional challenges include staff shortages, difficulties attracting and retaining qualified workers, and, in some parts of these sectors, lower average educational requirements. Common examples include healthcare and social care, emergency services, construction and heavy industry, transportation and logistics, and segments of trade, IT, finance, education, and social services (11, 12).

The concept of high-demand sectors is not formally established or consistently defined in research literature. Researchers more commonly describe specific occupations or work environments, rather than entire sectors, as “high-demand,” often drawing on frameworks such as the Job Demands–Resources (JD-R) model to explain how work characteristics shape health and performance (13, 14). Transport, health and social care, construction and retail trade are illustrative examples of such high-demand work environments. These sectors are of particular interest because they are critical to society, face persistent labor shortages, involve consistently high physical and/or emotional demands, and reflect different patterns of organizational conditions and risks. Accordingly, high-demand sectors in this study are defined by the nature of the work and its sustained physical and/or emotional demands, rather than by deficiencies in organizational conditions. Organizational-level health-promoting factors, often conceptualized as structures, processes, leadership practices, cultural norms, and policies that contribute to workplace wellbeing and organizational functioning, have received increasing attention. Prior studies emphasize organizational factors such as approachable and supportive leadership, clear and open communication, meaningful employee involvement, systematic work environment management routines, and the use of shared goals and core values as important contributors to employee wellbeing, reduced sickness absence, and organizational resilience (15–19). Although organizational-level health-promoting factors are relevant in all sectors, they are particularly important in high-demand sectors, where employees are exposed to substantial physical, and emotional demands. In such settings, organizational structures and practices may play a decisive role in buffering the impact of these demands and supporting employees’ health, work ability, and long-term participation in working life. Importantly, healthy organizations are not confined to low-risk sectors. A representative study of Swedish companies identified organizations with low levels of long-term sickness absence also in demanding sectors traditionally characterized by high sickness absence (15). Earlier high-demand sector-specific studies demonstrate the importance of organizational structures in shaping employee health. In healthcare, Åkerström et al. (17) identified five categories of organizational health-promoting factors: work scheduling, operational design and work methods, ergonomic conditions, working conditions and personnel policies, and the organization’s ethical environment. These findings underscore the importance of clear work processes, ethical climates, and sustainable scheduling practices for employee health and reduced sickness absence.

Beyond healthcare, organizational-level factors have also been shown to play an important role in other high-demand sectors. In construction, organizational conditions are particularly significant because work often takes place in temporary, multi-employer environments characterized by complex responsibility structures and high safety risks (20). The literature highlights management commitment, clear communication, employee involvement, safety management systems, and a strong safety culture as key factors in creating safer and more sustainable working conditions in the sector (21, 22). In trade and transport, practices such as fair personnel policies, participatory structures, and supportive supervision may be particularly important in contexts characterized by time pressure, irregular schedules, and high work demands (23, 24). Taken together, these findings suggest that organizational-level health-promoting factors are essential for enabling sustainable and healthy working conditions across high-demand sectors (11).

Although much of the existing research is sector-specific or focused on individual-level determinants, less attention has been paid to how organizational conditions support employee health across high-demand work environments. Given the complex organizational pressures within these sectors, greater knowledge is needed regarding the organizational-level health-promoting factors that contribute to the development and maintenance of healthy organizations over time.

The JD-R model (13, 14) offers a useful theoretical lens for understanding high-demand environments by proposing that high job demands can lead to strain unless balanced by sufficient resources, such as autonomy, social support, and organizational clarity. The JD-R framework extends beyond a risk-oriented perspective by also recognizing the positive role of workplace resources. It therefore integrates work performance, emphasizing how a well-functioning balance between demands and resources can benefit both employees and organizations. In high-demand sectors, the challenge is often not the presence of demands themselves, but rather the absence of buffering resources and sustainable organizational structures. Although the model is often applied at the individual or team level, it also provides a framework for examining organizational-level conditions (19).

The aim of this study was to deepen the understanding of organizational-level health-promoting factors by exploring experienced managers’ perspectives and visions regarding the fostering of healthy work environments in physically and/or emotionally demanding sectors.

## 2 Methods

This exploratory qualitative study was based on semi-structured, individual, digital interviews with managers working in physically and/or emotionally demanding sectors. Transport, health and social care, construction, and retail trade were selected as analytically relevant examples of private-sector work environments characterized by combinations of differing work organization, risk profiles, high physical, emotional, and/or temporal demands, recruitment and retention challenges, and substantial implications for employee health and organizational sustainability.

Ethical approval was obtained from the Swedish Ethical Review Authority (Dnr 2022–04798-01). The study was conducted in accordance with established ethical standards. All participants received written information about the study and provided written informed consent prior to participation. All research material and personal data were stored in a secure location, according to the General Data Protection Regulation (GDPR) at Uppsala University (2023).

This study followed the Consolidated Criteria for Reporting Qualitative Research (COREQ) to ensure transparency and rigor in reporting (25).

### 2.1 Participants and recruitment

Inclusion criteria for participation in the study were that individuals held a managerial position (e.g., operational manager, HR manager, or equivalent) in private-sector organizations with more than 50 employees, located in central Sweden, and operating within one of the following sectors according to the Swedish Standard Industrial Classification (SNI 2007): transport (SNI 49), health and social care services (SNI 87), construction (SNI 41–43), or retail trade (SNI 47). The aim was to recruit participants from approximately five organizations per sector. The target of approximately five interviews per sector was set to secure cross-sector breadth while maintaining feasibility within a qualitative design. The intention was not to make a sectoral comparison, but to ensure sufficient representation from each sector to identify recurring patterns across different physically and emotionally demanding sectors.

Recruitment took place between March and May 2023. A purposive sampling approach was initially employed. Potential organizations were identified through searches in a publicly available Swedish company register using the above criteria. The search yielded 22 companies in transport, 73 in construction, 17 in health and social care services, and 30 in the retail trade sector. These organizations were contacted via email, either directly to relevant managerial staff when contact details were available or via the general company email address. The invitation email stated that we were recruiting managers to participate in an interview study. The study sought to explore perspectives among managerial representatives regarding work practices and organizational structures that foster of healthy work environment. Follow-up telephone calls were made to non-responding organizations. In the recruitment phase, potential participants received an information describing the aim of the study, the nature of their participation, and how the data would be used.

Initial contact resulted in positive replies from three transport companies, two construction firms, four organizations in health and social care services, and two retail trade companies. However, one organization in health and social care services did not respond to interview scheduling requests. Because the target of approximately five organizations per sector was not fully met through the initial recruitment procedure, a snowball sampling strategy was subsequently applied. Interviewed managers were asked to recommend other eligible managers within their sector. The lower organizational size boundary was also pragmatically adjusted from more than 50 to more than 40 employees in order to include a sufficient number of organizations within the selected sectors.

Ultimately, representatives from five construction organizations, five health and social care organizations, six transport organizations (including two participants from the same company), and three retail trade organizations agreed to participate. During the interview process, three organizations (one each from construction, transport, and health and social care services) canceled their scheduled interviews and did not respond to repeated follow-ups. This resulted in a final sample of 16 managers representing 15 distinct organizations. Characteristics of the participating organizations and managers are presented in Table 1.

**Table 1:** Characteristics of participating organizations and managers.

Organizations					Managers			
Company number	Number of employees	Sector*	Geographical location**	Business activity	Title	Work experience in sector (Years)	Sex (M/F)	Age
1	70	T	C	Haulage company, bulk transport	HR and Health & Safety manager	18	F	50
2	300	T	C	Haulage company, freight transport	Terminal manager	26	M	44
3	225	T	C	Courier transport, freight transport, environmental transport	HR manager	12	F	48
4***	600	T	S	Passenger ferries	Deck officer	28	M	48
5***	600	T	S	Passenger ferries	Senior master	35	M	52
6	445	HS	C	Healthcare staffing	CEO	19	M	45
7	1,000	HS	C	Home care, residential care and home nursing	Regional manager	15	F	50
8	100	HS	N	Residential care and daily activities	Facility manager	30	M	63
9	63	C	N	Contracting, construction services and own house factory	Site manager	35	M	57
10	170	C	C	Developing and managing swimming and wellness facilities	Project manager	37	F	54
11	57	C	C	Construction in facade work, new and renovations	CEO	2	M	48
12	200	C	C	Building and civil engineering works	Health and Safety responsibility	6	M	43
13	500	C	C	Construction company, building and civil engineering	HR manager	3	F	39
14	80	RT	C	Hypermarket	Hypermarket manager	12	M	38
15	40	RT	S	Large supermarket	Store manager	16	M	36
16	40	RT	C	Builders’ merchants	Branch manager	15	M	58

*Sector: Transport sector (T), Health and social care services (HS), Construction industry (C), Retail trade sector (RT). **Geographical location: Northern Sweden (N), Central (C), Southern (S). *** Same organization.

The organizations varied in size, ranging from 40 to 1,000 employees. Within these organizations, all but one participant held leadership roles with staff responsibility. Participants occupied various managerial positions, including senior roles such as regional manager, HR manager, and health and safety manager. One participant was responsible for both human resources and health and safety. Two participants represented the same organization. The final sample comprised five women and 11 men, with a mean age of 48 years and an average of 18 years of work experience in their respective sectors. All participants possessed both sector-specific knowledge and experience working in physically and/or emotionally demanding work environments. Participants also expressed a strong interest in sharing their perspectives on how organizational practices could be improved to promote long-term sustainability and healthier work environments.

### 2.2 Data collection

Sixteen individual semi-structured interviews were conducted digitally via Zoom between August and November 2023. Each participant was interviewed on a single occasion, and only the participant and the researcher were present. Each interview lasted approximately 90 min and was conducted by one of two experienced interviewers: a psychologist and PhD candidate (ÅS) or an ergonomist and PhD (KE). The interviewers had no prior relationship with the participants before study commencement. The interviews followed an interview guide addressing key aspects of the work environment considered important for employee health and organizational sustainability. Topics included leadership, communication, work environment management, work-life balance, employee retention, sick-leave management, recruitment and onboarding, employee participation, skills development, and long-term work ability (see Appendix for the full interview guide). The interview guide was developed based on the authors’ previous research and the broader literature on organizational-level determinants of healthy work environments and psychosocial working conditions (3, 15, 16). The guide was pilot-tested during the first interview; however, no modifications were required.

Follow-up questions were asked as needed to deepen understanding and ensure that participants’ perspectives and visions were accurately captured. All interviews took place during regular working hours, the interviews were audio-recorded and transcribed verbatim, resulting in approximately 245 pages of single-spaced text.

### 2.3 Reflexivity of the research team

The research team comprised researchers with complementary professional and disciplinary backgrounds. ÅS is an organizational psychologist and PhD candidate with previous research experience concerning health-promoting organizational factors, primarily in the public sector. FM is a researcher in business administration whose research focuses on organizational behavior, communication, leadership, work environment, organizational development, and management control. KE is a researcher in ergonomics and human factors, with a research focus on work environment and occupational health. All three researchers have experience with qualitative research methods, and all have worked with organizations in the assessment and development of work environment conditions.

None of the researchers had previously studied the participating sectors from an organizational-level perspective focusing on health-promoting factors. The researchers therefore brought relevant knowledge of organizational health, leadership, communication, ergonomics, and work environment issues, while not having sector-specific research interests or pre-existing relationships with the participating organizations that could be expected to determine the findings.

During the interviews, the researchers used open-ended questions and follow-up prompts to encourage participants to describe their own experiences and views. Their professional backgrounds may nevertheless have influenced which issues were explored in greater depth and how participants’ statements were understood. The interviewers were aware of this possibility and sought to distinguish participants’ accounts from their own interpretations through follow-up questions, repeated reading of the transcripts, and discussion within the research team.

### 2.4 Data analysis

Interview data were analyzed using thematic analysis following the approach developed by Braun and Clarke (26), with NVivo 12 supporting data management, coding, and retrieval (27).

The analysis began as an inductive, data-driven process, in which codes were generated closely from participants’ accounts rather than being guided by a predefined analytic framework. Although the interview guide covered broad domains relevant to organizational health promotion (e.g., leadership, communication, and occupational health and safety), these domains were used to steer questioning and ensure breadth in the material, not as predetermined coding categories. As coding progressed, the analysis became increasingly abductive: the researchers iteratively moved between the empirical material and relevant theory on organizational health-promoting factors to refine and interpret emerging patterns (28). Thus, the analysis combined inductive coding of meaning units with theoretically informed interpretation during theme development and refinement, and no fixed, pre-identified thematic structure was used to structure the dataset from the beginning.

Following Braun and Clarke’s six phases, the process included familiarization with the data, initial coding, theme development, theme review, theme definition and naming, and report writing (26).

#### 2.4.1 Familiarization with the data

All interviews were transcribed verbatim and read repeatedly by two authors (ÅS and KE), both experienced in qualitative methods, to gain immersion in the material and develop an overall understanding of the participants’ accounts. During this phase, the authors made analytical notes on recurring issues, preliminary patterns, and material relevant to the study’s aim. At this stage, the analysis remained inductive and focused on participants’ explicit meanings in the data. During the analysis, all interviews were treated as a single managerial dataset, with no distinctions made between organizations, sectors, or managerial roles, as the aim was to identify shared patterns in how organizational-level health-promoting factors were conceptualized across the dataset rather than to compare perspectives across groups.

#### 2.4.2 Generation of initial codes

To strengthen analytical rigor, ÅS and KE independently coded the same two transcripts at the outset of the analysis. Coding focused on meaningful units of text relevant to the research question, and codes were generated closely from the data. The aim during this phase was to remain open to the breadth and variation of the material and to code inclusively rather than narrowing the analysis too early. After comparing and discussing their initial coding, the two authors established a shared understanding of the coding approach. The remaining transcripts were then divided between the two authors and coded individually.

#### 2.4.3 Searching for themes

After coding, the relationships among the codes were examined and grouped into broader units of meaning. As the large number of inductive codes became increasingly difficult to manage descriptively, the analysis entered a more explicit abductive phase. During this phase, theory on organizational-level health-promoting factors served as a sensitizing framework for interpreting emerging patterns—not to impose predetermined categories, but to deepen understanding of the data. Initial codes were organized into broader candidate themes and sub-themes reflecting recurring patterns across the dataset.

#### 2.4.4 Reviewing and refining themes

ÅS and KE compared their thematic structures, merged similar clusters, discussed discrepancies, and refined the candidate themes through repeated movement between coded extracts, sub-themes, and the full transcripts. The review process occurred at two levels: assessing whether the data within each theme formed a coherent pattern and evaluating whether the themes adequately represented the dataset as a whole. Through this process, some sub-themes were merged, others were divided, and the boundaries between the themes and sub-themes were clarified.

#### 2.4.5 Defining and naming themes

Once a stable thematic structure was established, all authors participated in refining and agreeing on the final themes and sub-themes. The researchers’ different perspectives were used to question, refine, and expand the emerging interpretations. Rather than seeking to eliminate researchers’ pre-understandings, themes were made explicit and critically reflected upon throughout the analytical process. The reflective process supported the development of themes that were grounded in the empirical material while allowing them to be interpreted in relation to relevant organizational health theories during the later stages of the analysis. Each theme was defined according to its central organizing idea and its relevance to the study’s aim. Theme names were selected to be both interpretive and grounded in the empirical material, while relationships between the themes and sub-themes were clarified to create a coherent analytical narrative.

#### 2.4.6 Producing the results report

During the final phase, the analysis was written as a coherent, analytically grounded account of the findings. Representative quotations were used to illustrate each theme and to connect the interpretations to the data. Overall, the analytical process was primarily inductive at the outset and became increasingly abductive in the later stages, allowing the findings to remain grounded in participants’ experiences while also being interpreted in relation to theories of organizational health promotion.

## 3 Results

The results are presented in four themes: (1) the need for a well-functioning, integrated occupational health and safety management system, (2) the need to strategically develop organizational attractiveness across the employee lifespan, (3) enabling present and supportive leadership focused on employee wellbeing, and (4) the importance of effective communication structures. The analysis also yielded 10 sub-themes. The themes were reflected across all included sectors, although specific examples sometimes took sector-specific forms. Table 2 provides an overview of the themes and sub-themes.

**Table 2:** Themes and sub-themes.

Themes	Sub-themes
The need for a well-functioning, integrated occupational health and safety management system	Building safety and risk awareness throughout the organization Promoting learning from best practices and peer organizations Engaging employees through multiple approaches to occupational health and safety improvement
The need to strategically develop organizational attractiveness across the employee lifespan	Adopting a lifespan perspective on recruitment, retention, and the value of senior employees Creating structured onboarding processes that support integration and safety Enabling competence development and flexible accommodation throughout the employment life cycle
Enabling present and supportive leadership focused on employee wellbeing	Being proactively present and demonstrating care for employee wellbeing and safety Fostering involvement, teamwork, and employee participation
The importance of effective communication structures	Establishing efficient information-sharing and feedback channels Fostering an open and supportive communication climate

Overall, the analysis showed that managers across the included high-demand sectors understood a healthy and sustainable organization as one in which care for employees is embedded in organizational structures, routines, and leadership practices rather than left to individual goodwill. Across the interviews, employee health, workforce sustainability, and organizational functioning were consistently linked to an overarching organizational orientation characterized by safety, attentiveness, and long-term responsibility in everyday work. This pattern can be described as an organization-wide culture of care, expressed through systematic occupational health and safety management, sustainable employment practices across the career span, supportive and present leadership, and open communication structures.

### 3.1 Theme 1: the need for a well-functioning, integrated occupational health and safety management system

A first central theme concerned the importance of occupational health and safety (OHS) management being systematic, integrated, and embedded within everyday organizational functioning. Managers consistently emphasized that OHS could not be reduced to formal compliance or isolated procedures; instead, it needed to permeate daily work, decision-making, and organizational culture.

#### 3.1.1 Building safety and risk awareness throughout the organization

Managers described safety and risk awareness as foundational to a healthy organization. Health-promoting work was understood as requiring more than protective equipment or written policies. Instead, respondents emphasized the importance of cultivating a shared culture in which health, wellbeing, and risk awareness are openly discussed across occupational groups and hierarchical levels.

One manager explained:

*“You think about health and well-being both outside work and at work. And you create a culture where people talk about it more openly—both with site managers, supervisors, and the workers at the same time. That is what has had the greatest effect, because it is not enough just to provide the right equipment.”* (Participant 12, construction)

This illustrates how safety was framed as a collective organizational mindset rather than a purely technical matter. Regular structures such as morning meetings, toolbox talks, and staff meetings were described as important arenas for making safety visible, normalizing discussions of risk, and reinforcing shared responsibility.

#### 3.1.2 Promoting learning from best practices and peer organizations

A second sub-theme concerned the importance of organizational learning. Managers described how OHS was strengthened through both internal and external learning, for example, through participation in industry networks, use of support functions, and adopting practices from other departments or peer organizations. One manager described the value of sharing experiences between organizations:

*“We can share good examples between stores. These days, there are so many networks to work with in all kinds of areas—whether it concerns HR issues, fruit-related matters, or really anything at all. There is a great deal of support available, as well as many opportunities to share good examples.”* (Participant 14, retail trade)

Best practices were viewed as valuable resources that could be adapted to local needs and operational realities. One respondent referred to another department as a local example of successful safety work:

*“Yes, we have a really skilled machinery department that is very far ahead in this… They’ve done a great job reducing injuries and getting people involved… After every morning meeting and toolbox meeting they have, they write down what they talked about. Everyone also signs to show they were there and heard it.”* (Participant 4, transport)

Here, documentation, follow-up, and visible participation were portrayed as integral components of a mature and accountable safety system. Learning also involved preserving experiential knowledge from long-serving and retired employees. Such accounts suggest that systematic OHS management is strengthened when organizations function as learning systems in which knowledge is continuously accumulated, shared, and retained over time.

#### 3.1.3 Engaging employees through multiple approaches to OHS improvement

Managers further emphasized that effective OHS management depends on employee participation through both multiple formal and informal approaches. Surveys, meetings, collaboration with safety representatives, and continuous dialogue were described as important mechanisms for identifying problems and improving working conditions. The use of support functions was also emphasized, including human resources, occupational health and safety consultants, occupational health services, and industry networks. Employees were not viewed merely as recipients of managerial initiatives but as indispensable contributors to identifying problems and developing solutions. As one manager stated:

*“Not just pointing out the problem, but also asking employees what they think the solution is. They’re the ones who know the problem best, after all… So, it’s about inviting people in and really listening… Everything should come from the ground up. They know their own workplaces best.”* (Participant 16, retail trade)

This excerpt illustrates how health-promoting work environment management was understood as participatory and dialogic. Involving employees enhanced both the practical relevance of improvement efforts and their legitimacy within the organization. Another manager highlighted this integrated approach, emphasizing how employee involvement was embedded within broader organizational systems.

*“We don’t really separate work environment, quality, and environmental issues. We work with all of it together because our system is integrated. And you see the same thing there—people get really engaged when you go out and ask them: ‘This happened—do you have any ideas? What do you think caused it?”* (Participant 1, transport)

### 3.2 Theme 2: the need to strategically develop organizational attractiveness across the employee lifespan

The second theme concerned how managers described organizational attractiveness as a strategic, long-term, and lifespan-oriented responsibility. Rather than focusing solely on recruitment, respondents emphasized the importance of creating supportive conditions across the full employment trajectory, from pre-boarding and onboarding to competence development, retention, flexible accommodation, and post-retirement engagement. Healthy organizations were thus portrayed as places that deliberately create conditions for sustainable working lives over time.

#### 3.2.1 Adopting a lifespan perspective on recruitment, retention, and the value of senior employees

Managers frequently adopted a lifespan perspective, emphasizing the importance of attracting younger employees while also retaining older workers and valuing retirees as essential organizational resources. Employer attractiveness was seen as particularly important in sectors characterized by labor shortages, and a positive reputation was described as emerging from everyday organizational practices such as safety, respectful treatment, and care. Organizations sought to attract younger workers through internships, apprenticeships, and a strong workplace reputation. Employees were also portrayed as ambassadors who communicated this reputation externally, thereby supporting recruitment. One manager noted:

*“We have worked a lot with our employees so that those who enjoy working at the company create positive word of mouth externally. They want to talk about their company. It is connected to the work environment and job satisfaction, that you feel truly seen at work.”* (Participant 12, construction)

At the same time, older workers were viewed as crucial for continuity, mentoring, and staffing flexibility. As one participant stated:

*“And I’ve never been in an industry where there are so many retirees who still want to work. We wouldn’t manage a single day without them.”* (Participant 3, transport)

Senior employees and retirees were described as contributing stability, reliability, practical wisdom, and mentoring capacity. Their role was often framed as a strategic asset, with their competence and dependability viewed as essential for sustaining daily operations. Incentive structures, including higher wages for employees transitioning into additional staffing roles, were implemented to facilitate continued employment. Flexible task allocation and working hours were also identified as important conditions for prolonging working life, for example by offering less or more demanding routes and working conditions in the transport sector.

#### 3.2.2 Creating structured onboarding processes that support integration and safety

A structured onboarding process was described as essential for both safety and retention. Managers emphasized the importance of clear role expectations, documented routines, practical guidance, and access to experienced colleagues. Several components of effective onboarding were identified. Pre-boarding initiatives were viewed as beneficial in preparing employees prior to their start date. Clear employment contracts, role descriptions, and defined responsibilities were considered foundational. Assigning experienced colleagues for shadowing during the initial period was described as an effective way to support learning and build confidence. New employees were also expected to engage in mandatory training.

Onboarding was framed not merely as orientation, but as a structured process for building competence, confidence, and psychological safety. Digital tools were increasingly used to structure and quality-assure the process, including applications that enabled daily feedback from new employees and allowed managers to monitor progress. Additional measures included mentorship programs, employee handbooks, and scheduled follow-up meetings. Evaluation and follow-up, often conducted through interviews with newly hired employees, were considered essential.

In sectors with a substantial proportion of employees with foreign backgrounds, onboarding processes were adapted to account for differences in language proficiency and cultural context. Clear communication and explicit encouragement to ask questions were described as particularly important. In some cases, structured practice environments were provided before employees entered real work situations—for example, through the use of a training apartment in home care services, allowing employees to rehearse tasks in a controlled setting. One participant explained:

*“I think we need to work more with having a buddy or a mentor… making sure it is structurally documented, that there are staff handbooks, and that you always know where to find information.”* (Participant 6, health and social care)

This suggests that effective onboarding depended on both relational support and accessible organizational systems. Managers also linked careful onboarding to injury prevention and long-term efficiency:

*“A lot of workplace accidents could be avoided if people had more information, more internal training, and proper introductions… If we do things properly from the start, we’ll have fewer problems later.”* (Participant 1, transport)

Thus, onboarding was understood as an early preventive investment that could strengthen retention, reduce accidents, and facilitate safe integration into demanding work environments, thereby supporting sustainable working lives over time.

#### 3.2.3 Enabling competence development and flexible accommodation throughout the employment lifespan

Managers expressed a clear intention to support employees’ long-term sustainability through continuous competence development and flexible accommodation across the entire employment lifespan. Competence development was seen as essential not only for maintaining quality and safety standards, but also for promoting employee motivation and professional engagement over time. This included technical training, ergonomics, and work methods, as well as softer skills such as communication, active listening, and teamwork. Organizations increasingly invested in structured solutions such as digital competence portals with web-based courses, trainee programs, and internal career pathways. One manager described an ongoing initiative as follows:

*“We’re working right now on developing a competence portal with a whole range of web-based training courses that our employees can and should take. It’s part of pre-boarding, onboarding, and development later on. It’s going to be a huge boost for our employees.”* (Participant 3, transport)

At the same time, participants highlighted the need for flexibility and individualized accommodations, particularly for senior employees and those with reduced work capacity. Flexible task allocation, adjusted working hours, and modified assignments were described as important means of prolonging working life and retaining valuable experience. Senior employees and retirees were often portrayed as strategic assets who continued to contribute on their own terms. One participant explained:

*“There are a lot of retirees who choose to keep working, but on their own terms… I can work one day a week, or two days a week… We usually say they are worth their weight in gold.”* (Participant 3, transport)

This combined focus on competence development and flexible accommodation was understood as a deliberate organizational strategy. By investing in ongoing learning while adapting working conditions to individuals’ changing capacities, organizations aimed to preserve competence, reduce health risks, strengthen retention, and support sustainable working lives.

### 3.3 Theme 3: enabling present and supportive leadership focused on employee wellbeing

Managers described leadership as a central organizational practice embedded in everyday operations, rather than an individual personality trait. They emphasized the need for leaders who are physically and relationally close to daily operations in order to demonstrate genuine care, build trust, and support employee wellbeing in high-demand environments.

#### 3.3.1 Being proactively present and demonstrating care for employee wellbeing and safety

Participants emphasized the importance of leaders being visible and accessible in the work environment. Proactive presence enabled early identification of risks, more timely responses to emerging issues, and stronger personal connections with employees. Managers described how leaders should be present in daily operations, engage in informal interactions such as coffee breaks, and maintain personal contact during situations such as sickness absence. This closeness was understood as a concrete expression of care. One manager illustrated the value of everyday presence:

*“As a manager, you should be at the office early enough to sit down and have coffee with them before they head out.”* (Participant 7, health and social care)

Another stressed the importance of being physically present in the workplace:

*“Get out to the workplace. It doesn’t matter whether it’s out on the scaffolding, in the site cabin, at a conference, or in the parking lot—be there. Stand with them. Then communication won’t be a problem.”* (Participant 11, construction)

Care was further demonstrated through individualized support and small gestures of appreciation, such as providing breakfast after particularly demanding periods. Participants framed this as an organizational stance:

*“There should always be a sense of care. We care a lot for one another.”* (Participant 1, transport)

This form of leadership was described as dependent on organizational preconditions, including access to support functions and structured leadership development, and was understood as a concrete expression of care in practice.

#### 3.3.2 Fostering involvement, teamwork, and employee participation

Managers described a shift away from top-down decision-making toward more participatory approaches that promote shared ownership and team cohesion. Leaders were expected to create conditions in which employees felt involved in shaping their work practices. Participants emphasized that successful implementation often occurred when employees were engaged in generating ideas. One manager explained:

*“When there is a need for changes, it is important to actually involve the employees. ‘Look… we need to review this and we need to make changes. If you have good suggestions on how it can be done - share them.’ So that you incorporate them [the employees].”* (Participant 7, health and social care)

Another participant highlighted the importance of keeping employees informed and explaining resource constraints when suggestions could not be implemented immediately. This participatory approach was understood as essential for fostering engagement, mutual support, and safety in demanding work contexts.

### 3.4 Theme 4: the importance of effective communication structures

In work settings characterized by shift work, dispersed teams, and high operational risk, managers emphasized the need for communication structures that are timely, reciprocal, and psychologically safe. Communication was not viewed merely as the transfer of information, but as a foundation for trust, learning, safety, and participation.

#### 3.4.1 Establishing efficient information-sharing and feedback channels

Managers highlighted the challenge of reaching all employees in continuous operations and emphasized the need to adapt communication to operational realities. Direct, personal channels were preferred over passive ones. Participants described how information about upcoming tasks could be shared via digital collaboration platforms, while urgent matters were best communicated through phone calls or text messages. One manager noted:

*“We try to keep each other informed. It’s difficult because we work in shifts around each other… put it on Teams so everyone can see what needs to be done.”* (Participant 5, transport)

Another participant was more explicit about preferred channels:

*“But the website is completely useless. Almost as useless is email… otherwise, a direct phone call is incredibly effective. Text messages and phone calls.”* (Participant 11, construction)

These accounts illustrate how effective communication structures were understood not as neutral administrative tools, but as practical systems that must align with the temporal and spatial conditions of high-demand work in order to function effectively. Feedback channels were equally important, as they enabled both operational adjustments and ongoing development.

#### 3.4.2 Fostering an open and appreciative communication climate

Managers emphasized the importance of cultivating an open and appreciative communication climate in which employees feel safe to raise concerns, suggest improvements, and make mistakes without the fear of negative consequences. At the same time, leaders were expected to provide both developmental feedback and explicit recognition of employees’ efforts. One participant described the importance of openness:

*“I have a permissive culture. I let people try things and make mistakes. But also, they can come to me and say pretty much anything, and I won’t tear them down for it.”* (Participant 15, retail trade)

Another manager emphasized the importance of responding to ideas raised by employees:

*“If someone gathers the courage and energy to speak up about something, you also need to respond to it quickly. We encourage our managers to at least give a thumbs-up—to show that the feedback has been received… because it is valuable for people to feel that their voice is heard.”* (Participant 13, construction)

Another participant explained the underlying purpose of feedback:

*“What’s the whole point of giving feedback? It’s to help the person grow, improve the business, or make a positive change. If that’s not there at the core, then it’s just nonsense.”* (Participant 16, retail trade)

This combination of openness, psychological safety, and appreciative recognition was seen as essential for building trust, enabling organizational learning, and supporting employee wellbeing and motivation in high-demand sectors.

## 4 Discussion

This study advances the understanding of organizational-level health-promoting factors in physically and emotionally demanding sectors by foregrounding managers’ perspectives on what enables healthy and sustainable work environments. Across transport, health and social care, construction, and retail trade, the findings suggest that healthy organizations are not primarily created through isolated individual supports but through systematic organizational conditions that shape everyday work. This interpretation aligns with broader research emphasizing that organizational commitment to employee wellbeing and supportive leadership are key determinants of healthy workplaces. The results also confirm previously established organizational factors related to health (15, 16), thereby contributing to the growing body of research on organization-level determinants. Four interrelated themes were identified: an integrated occupational health and safety management system, lifespan-oriented organizational attractiveness, present and supportive leadership, and effective communication structures. Taken together, these themes indicate that employee wellbeing in high-demand contexts depends on how organizations structure work, allocate responsibility, communicate priorities, and enact care through leadership and everyday practices.

A central contribution of the study is the identification of an overarching organizational culture of care that connects all four themes. Although the overarching themes were recognizable across sectors, their practical enactment varied depending on sector-specific work organization, mobility, safety risks, and staffing conditions. Managers described health promotion as embedded in structures, routines, and decision-making rather than as an optional or individualized add-on. This interpretation is consistent with organizational perspectives emphasizing that upstream conditions, such as policies, procedures, and management priorities, shape both job demands and available resources (17, 29). The findings therefore support arguments that sustainable occupational health practice should prioritize organizational interventions over individual coping strategies, consistent with the hierarchy of controls in occupational safety and health (8).

The first theme—a well-functioning, integrated occupational health and safety management (OHS) system—underscores that OHS work is most effective when embedded in daily operations. Managers did not describe safety as a separate compliance task but as a cultural and operational orientation requiring shared awareness, ongoing dialogue, and mechanisms for learning. This interpretation resonates with multilevel safety climate research showing that organizational priorities are translated into local practices and behaviors through shared perceptions of what is genuinely valued (30). It also reflects broader notions of psychosocial safety climate, in which senior management’s commitment to worker health establishes the context in which demands and resources are configured (31). The findings therefore suggest that systematic OHS management may operate not only as a formal requirement but also as a health-promoting organizational resource in its own right.

The emphasis on learning from best practices and peer organizations further extends previous literature. Whereas prior studies have often focused on internal safety procedures, managers in this study also highlighted the importance of cross-unit learning and the preservation of senior workers’ experiential knowledge. This underscores the dynamic character of organizational health promotion, as healthy organizations function as learning systems that identify, retain, and circulate practical knowledge. These findings echo Miño-Terrancle et al.’s (32) argument that preventive learning requires an integrated, collaborative, and systemic approach, while also connecting to broader organizational learning perspectives that link adaptive capacity to resilience in changing work environments.

The second theme—strategic, lifespan-oriented organizational attractiveness—adds a novel dimension to the literature on healthy organizations in high-demand sectors. Managers framed attractiveness not merely as recruitment branding but as a long-term organizational responsibility spanning pre-boarding, onboarding, competence development, retention, accommodation, and post-retirement engagement. This broader temporal perspective is particularly important in sectors facing labor shortages and aging workforces, suggesting that health promotion and workforce sustainability are deeply intertwined. Earlier reviews have emphasized scheduling, work methods, and personnel policies as organizational determinants of healthcare workers’ health (17); the present findings extend this perspective by showing how managers across multiple sectors strategically connect employee wellbeing to long-term employability and organizational continuity.

The importance attributed to senior employees and retirees was particularly notable. Rather than being viewed primarily through a deficit lens, older workers were described as carriers of tacit knowledge, stability, and mentoring capacity, something that was particularly prominent among the interviews in the transport and construction sectors. This challenges assumptions that sustainable organizations focus mainly on early-career attraction and instead highlights the importance of age-inclusive organizational practices. Such practices may also mitigate competence loss in high-demand sectors. These findings reinforce arguments that organizational sustainability must be understood across the life course rather than solely in relation to current staffing needs.

The importance of structured onboarding was another aspect of organizational attractiveness. Managers consistently linked well-documented onboarding to safety, confidence, and retention. This reflects an organizational understanding that integration into demanding work requires more than administrative orientation; it involves social inclusion, role clarity, and practice-based learning. In high-demand contexts, where errors can have serious consequences, onboarding may function as a preventive intervention by reducing uncertainty and enhancing competence and psychological safety. This complements existing research showing that the design of work processes and organizational support can either intensify or mitigate workplace strain (17, 33).

The third theme—present and supportive leadership focused on employee wellbeing—reinforces the importance of leadership as a core organizational mechanism for health promotion. Managers emphasized visibility, accessibility, and relational closeness to daily operations. Such leadership was not described in abstract or charismatic terms but in practical acts of attentiveness—being present on site, noticing strain, maintaining contact with the employee during sickness absence, and recognizing employees’ efforts. These findings are consistent with prior research identifying leadership as central to promoting health, safety culture, and productivity in changing environments (32, 34).

These findings are also consistent with the literature on safety culture and safety leadership in high-risk industries (35–37). Safety culture refers to the shared values, norms, and practices through which organizations signal priorities in everyday work, while safety leadership captures how managers influence safety-related attitudes and behaviors through visible commitment, communication, and role modeling. In organizations facing high physical and emotional demands, leadership that emphasizes safety, supports reporting and learning, and maintains close contact with day-to-day work can strengthen employees’ perceptions that wellbeing is a genuine organizational priority rather than a symbolic one. In this light, the present findings suggest that the organizational culture of care identified in this study may also be understood as a form of safety culture enacted through everyday leadership practices.

Importantly, the managers’ accounts indicate that supportive leadership is enabled or constrained by organizational conditions. Present leadership requires reasonable spans of control, access to support functions, and organizational expectations that make such presence possible. This is theoretically significant, as it moves beyond treating leadership solely as an individual competency. Instead, it positions leadership as an organizationally situated practice, dependent on structures, resources, and decision latitude. This aligns with multilevel perspectives emphasizing that leadership behaviors both shape and are shaped by higher-order climates and systems (30).

The participatory aspects of leadership described by managers are also noteworthy. Employees were seen as crucial contributors to problem-solving, particularly in OHS and operational improvements. This aligns with evidence that participation, communication, and shared ownership are central health-promoting organizational factors (18). It also supports prior findings that a preventive culture requires commitment from both management and workers, rather than being imposed in a top-down manner (32).

The fourth theme—effective communication structures—illustrates the operational dimensions of organizational health promotion. Overall, communication was highlighted as important across the sectors, although communication structures took different forms in dispersed transport operations than in more site-bound organizations. In sectors characterized by shift work, mobile work, dispersed teams, or time pressure, communication was understood as a critical infrastructure for safety, coordination, and a sense of belonging. The interviewed managers within these sectors, especially transport, construction, and health and social care, stressed the need for communication channels that were timely, direct, and adapted to operational realities. The importance of communication aligns with earlier literature identifying communication as a central component of healthy workplaces and preventive cultures (18, 32).

Equally important was the emphasis on an open and appreciative communication climate. Managers described the need for employees to feel safe raising concerns, admitting mistakes, and offering suggestions. Such accounts align with the concepts of psychological safety and psychosocial safety climate, in which open communication and non-punitive responses to risk are foundational for learning and wellbeing (38). In emotionally and physically demanding sectors, where silence may increase both hazard exposure and emotional strain, such communication climates may be especially consequential.

From a theoretical perspective, the findings can be interpreted through the Job Demands–Resources model (JD-R) (13, 14). The sectors represented in this study were characterized by persistently high demands, yet managers rarely framed them as the primary problem. Rather, risks to health were discussed in relation to the adequacy of organizational resources, including leadership presence, onboarding, staffing flexibility, communication, support systems, and learning structures. This supports the JD-R proposition that strain emerges not only from high demands but also from an imbalance between demands and resources. The findings also extend the model by showing how such resources are created and sustained at the organizational level, rather than only experienced at the individual level.

Relatedly, the concept of Psychosocial Safety Climate (PSC) highlights the organizational conditions that shape these demand–resource configurations. PSC refers to employees’ shared perceptions that organizational policies, practices, and procedures prioritize psychological health and safety and is primarily shaped by senior management’s commitment and values (Dollard & Bakker, 2010). Within the JD-R framework, PSC can be understood as an antecedent influencing both the level and quality of job demands and job resources (39, 40). A strong PSC is theorized to reduce harmful demands and strengthen resources such as autonomy, social support, and employee involvement, thereby helping explain why some organizations create more favorable psychosocial working conditions than others.

The study also has practical implications. First, it suggests that organizations in high-demand sectors should strengthen integrated and participatory OHS systems, rather than relying on reactive or fragmented approaches. Second, organizations should adopt a lifespan perspective in workforce strategy, recognizing onboarding, development, accommodation, and post-retirement engagement as interrelated elements of health promotion and organizational sustainability. Third, leadership development should focus not only on interpersonal skills but also on ensuring the structural conditions that enable present, supportive, and participatory leadership. Finally, organizations should design communication systems that are both operationally effective and psychologically safe.

### 4.1 Strengths and limitations

A major strength of this exploratory qualitative study is its exploration of managers’ perspectives on the organizational conditions they considered important for developing healthy and sustainable work environments in sector-specific contexts. Informed by Positive Occupational Health Psychology (6) and Positive Organizational Scholarship (41), the study focused on organizational-level health- promoting factors, with the aim of generating insights that may be relevant across workplaces. The multisectoral design, including transport, health and social care, construction, and retail trade, further enabled the identification of common organizational-level health-promoting factors across demanding work environments. Given the exploratory multisectoral design the aim was not to achieve information saturation within each sector, but to obtain sufficient breadth and variation to identify recurring organizational-level health-promoting factors across demanding work environments.

Recruitment was challenging and influenced the composition of the sample. The initial purposive sampling strategy was supplemented by snowball sampling, and the minimum organizational size was reduced. These pragmatic changes supported the recruitment of a sufficient number of participants across sectors but may also have introduced selection bias. Organizations or managers with a particular interest in work environment and employee wellbeing, or those who perceived their organizations as having relatively well-developed organizational conditions for supporting employee health, may have been more likely to participate. However, this is also consistent with the exploratory aim of the study, which was to identify and understand organizational-level health-promoting factors, rather than to estimate the prevalence of such factors or provide a representative picture of organizational conditions across high-demand sectors. Accordingly, the study was designed to explore what managers perceived to support employee health, rather than to identify organizational problems or risk factors.

An alternative approach would have been to recruit organizations with documented success in occupational safety and health (OSH) or employee wellbeing. However, this was not feasible because reliable and comparable information on organizational practices and outcomes was not available. Instead, organizations that met the inclusion criteria and were willing to participate were recruited. The findings should therefore be interpreted as managers’ perspectives on organizational-level health-promoting factors, rather than as evidence that the participating organizations were exceptionally successful. Participants occupied different managerial roles and hierarchical levels. This diversity was a strength, as it provided insights from multiple managerial perspectives. However, it may also have influenced the findings, since participants with different responsibilities may have been more likely to emphasize different aspects of organizational health promotion. For example, senior managers may have focused more on strategic priorities and organizational performance, first-line managers on daily operations and working conditions, and HR or occupational health and safety managers on formal systems, policies, and routines. The findings should therefore be understood as reflecting a range of managerial perspectives rather than a single managerial viewpoint. Because the aim was to identify shared organizational patterns rather than compare managerial roles, all participants were analyzed as a single dataset. Future research could explore how organizational-level health-promoting factors are perceived across different managerial levels and functions.

A further limitation is that all data were obtained from managers. Employees may experience organizational conditions differently and future studies should compare managerial and employee perspectives and examine how the identified organizational conditions are experienced and enacted in everyday work. At the same time, the exclusive focus on managers was consistent with the aim of the study, as managers have formal responsibility for shaping organizational structures, priorities, and working conditions, and therefore offer a perspective that is particularly relevant for understanding organizational-level health promotion.

In addition, all participating organizations were in the private sector, and the study did not systematically compare the sectors or distinguish private from public health and social care organizations. The findings may therefore not be equally transferable to public organizations operating under different institutional and organizational conditions.

The research team’s professional and disciplinary backgrounds also informed the study. The researchers have expertise in organizational psychology, business administration, ergonomics/ human factors, as well as experience in qualitative research and work environment assessment and development. The team approached the material with an interest in organizational-level conditions that promote employee wellbeing, sustainable work, and healthy organizational functioning. These perspectives sensitized the researchers to issues such as leadership, employee participation, communication, OSH management, and organizational learning. However, these concepts did not serve as predefined coding categories during the initial stages of analysis. Instead, the researchers remained open to both expected and unexpected aspects of the participants’ accounts. During the later abductive stages, the researchers discussed their interpretations collectively, and their different disciplinary perspectives were used to critically question, refine, and broaden the emerging interpretations before agreeing on the final themes.

The team’s interest in understanding organizational conditions that promote employee health nevertheless contributed to an emphasis on constructive and positive accounts, consistent with the study’s aim. This may have reduced attention to tensions, failures, or critical experiences. Social desirability may also have influenced participants’ descriptions of their organizations and managerial practices. The researchers therefore sought to remain attentive to variation, ambiguity, and possible contradictions in the material.

Finally, the qualitative and exploratory design does not permit statistical generalization. Its contribution lies instead in providing analytical depth and generating insights into organizational conditions that promote employee health for further investigation. The inclusion of several sectors supports potential transferability, but the relevance of the findings should be assessed in relation to the specific organizational and occupational context.

## 5 Conclusion

In conclusion, this study suggests that healthy organizations in high-demand sectors are characterized not by isolated supportive practices, but by an embedded organizational culture of care. This culture is expressed through integrated health and safety management, long-term workforce strategies, supportive and present leadership, and open communication. These findings reinforce the importance of addressing health promotion at the organizational level and contribute to a growing body of research demonstrating that sustainable work environments are built through deliberate structures, cultures, and managerial priorities, rather than relying on individual resilience alone.

## Data Availability

The datasets presented in this article are not readily available because data transcripts from interviews may be made available by the authors upon reasonable request and subject to ethical and legal considerations, including GDPR and confidentiality requirements. Requests to access the datasets should be directed to kristina.eliasson@medsci.uu.se.
