# Supplementary material for: Creating healthy organizations: an integrated culture of care in high-demand sectors—a qualitative study of private-sector organizations in Sweden
Source: Front Public Health. 2026 Sep 16;14:1888516. doi: 10.3389/fpubh.2026.1888516 (PMC13624139; doi:10.3389/fpubh.2026.1888516)
Supplement: Supplementary file 1 [file Data_sheet_1.docx]

**Supplementary material**

**Interview Guide**

You are welcome to provide specific examples from your organization, but also to reflect more broadly on how work in this area could be approached.

*Background***:** Age, education, job title, how long you have worked in your role, at the company, and in the sector, size of the company, number of employees.

- What do you see as the major challenges and opportunities for achieving a sustainable work life for employees in your sector?
- What are your thoughts on how to retain workers?
- What are your thoughts on how to prevent work-related ill health?
- Do you have any ideas about how a sustainable work life could be promoted for your employees?
- Do you see any particular challenges in the sector regarding occupational health and safety efforts?
  - How do you think workplace environment issues can be addressed?
  - How can external parties be utilized as support in your occupational health and safety efforts?
    - Do you have any experience with external collaboration on occupational health and safety efforts?
  - How can digital systems be used as support/help in occupational health and safety efforts?
  - How can employees be engaged/active in occupational health and safety efforts?
- What are your thoughts on how to work to reduce the number of sick leave in your sector?
- How do you think short-term sick leave can be reduced?
- How do you work with employees on long-term sick leave to support their return to work? Do you have any specific routines for this?
- Do you have any thoughts on how to identify employees who may be at risk of long-term absence?
- Do you have any ideas regarding workplace accommodations and whether

or how they can be implemented?

- Do you have any thoughts on how work life and personal life can be balanced in your sector?
- What are your thoughts on recovery and time for reflection?
- Do you have any thoughts on how employees can be engaged in the company's/industry's development and improvement efforts?
- How can employers influence employees’ perception that their work is meaningful?
- What are your thoughts on how to make use of all *employees' resources* throughout their working lives?
  - How can your sector support employees in working until the ages of 68-70?
  - How can situations be handled where an employee's physical and/or mental capacity no longer matches the demands required for the job? Accommodations?
- How can open *communication* between employees and managers be promoted in your sector?
- Are there specific communication methods/channels that work better/worse for you?
- How can you provide each other with feedback effectively?
- What are your thoughts on the type of *leadership* needed in your sector?
- How can you recruit to get the kind of leaders that the sector needs?
- What requirements should the sector have for leaders?
- What conditions are necessary in the organization to enable good leadership?
- How can you follow up on decisions made and ensure they are effective?
- How can you promote employees’ sense of being treated fairly in the workplace and by their managers?
- How can employees be supported in prioritizing tasks when there is a lot to do?

*Recruitment and Competence Development*

- What are your thoughts on what is important for successful recruitment?
- How can employees be effectively recruited within your sector?
- What are your thoughts on what is important in providing employees with a good introduction to the workplace?
- How can you become an attractive employer that employees actively seek out?
- Do you have any thoughts on how to work with competence development to ensure the skills needed in your sector?
- How can you better utilize the skills and competencies that individual employees possess?

Before we conclude, is there anything else you think is important to highlight in promoting a healthy and sustainable work life in your sector?
